# Non-Contact Assessment of Swallowing Dysfunction Using Smartphone Captured Skin Displacements

**DOI:** 10.3390/s23125392

**Published:** 2023-06-07

**Authors:** Nikyta Chesney, Prashanna Khwaounjoo, Maggie-Lee Huckabee, Yusuf Ozgur Cakmak

**Affiliations:** 1 Department of Anatomy, University of Otago, Dunedin 9016, New Zealand; 2 Rose Centre for Stroke Recovery and Research, University of Canterbury, Christchurch 8140, New Zealand; 3 Auckland Bioengineering Institute, University of Auckland, Auckland 1010, New Zealand; 4 Centre for Bioengineering and Nanotechnology, University of Otago, Dunedin 9054, New Zealand

**Keywords:** dysphagia, non-contact, phase-based Savitzky–Golay gradient correlation, image registration algorithm, larynx, penetration aspiration, residue, mobile phone, mobile health, remote monitorization

## Abstract

Early and accurate dysphagia diagnosis is essential for reducing the risk of associated co-morbidities and mortalities. Barriers to current evaluation methods may alter the effectiveness of identifying at-risk patients. This preliminary study evaluates the feasibility of using iPhone X-captured videos of swallowing as a non-contact dysphagia screening tool. Video recordings of the anterior and lateral necks were captured simultaneously with videofluoroscopy in dysphagic patients. Videos were analyzed using an image registration algorithm (phase-based Savitzky–Golay gradient correlation (P-SG-GC)) to determine skin displacements over hyolaryngeal regions. Biomechanical swallowing parameters of hyolaryngeal displacement and velocity were also measured. Swallowing safety and efficiency were assessed by the Penetration Aspiration Scale (PAS), Residue Severity Ratings (RSR), and the Normalized Residue Ratio Scale (NRRS). Anterior hyoid excursion and horizontal skin displacements were strongly correlated with swallows of a 20 mL bolus (r_s_ = 0.67). Skin displacements of the neck were moderately to very strongly correlated with scores on the PAS (r_s_ = 0.80), NRRS (r_s_ = 0.41–0.62), and RSR (r_s_ = 0.33). This is the first study to utilize smartphone technology and image registration methods to produce skin displacements indicating post-swallow residual and penetration-aspiration. Enhancing screening methods provides a greater chance of detecting dysphagia, reducing the risk of negative health impacts.

## 1. Introduction

### 1.1. Background

Dysphagia, a swallowing impairment, can occur as a result of many etiologies, including but not limited to stroke, neuromuscular and neurodegenerative diseases, traumatic brain injuries, and cancers of the head and neck [1]. Dysphagic patients are at an increased risk for mortality, co-morbidities, and adverse effects such as aspiration pneumonia, dehydration, malnutrition, and choking events [2,3,4]. Complications associated with dysphagia can lead to a significantly increased length of hospital stay, need for assistance, number of medical procedures, and health care costs [4,5]. The intimate relationship between eating and drinking in social and community settings can significantly impact the quality of life (QOL) in dysphagic patients with associations of anxiety, depression, and isolating behaviors [2,3,6].

Aspiration and post-swallow residue are arguably the most salient risk factors assessed when evaluating dysphagia. Aspiration, the entry of foreign material into the airway, is a primary risk factor for aspiration pneumonia and increases the risk of mortality following a stroke [7,8]. Pharyngeal residue, the post-swallow retention of material in the pharynx, is often located in the vallecula and pyriform sinuses [9]. The airway becomes open and unprotected post-swallow. Hence, the presence of residue increases the risk of aspiration as retained material can easily spill into the airway [10].

### 1.2. Current Dysphagia Evaluation Approaches

Early and accurate diagnosis of dysphagia is essential for effective treatment and preventing negative outcomes [11]. Assessments include different screens, clinical evaluations, and instrumental assessments such as videofluoroscopic swallowing study (VFSS), pharyngeal manometry, and fiberoptic endoscopic evaluation of swallowing (FEES) [12,13]. Dysphagia can go undetected without the incorporation of instrumental procedures, with drastic differences in prevalence rates of 36% without instrumental procedures and 81% when incorporating instrumental procedures [13]. Inaccurate diagnosis of dysphagia may increase the risk of adverse effects, particularly for silent aspirators, who are at a high risk of remaining undiagnosed [14].

VFSS, the gold standard of dysphagia assessment, has observational properties, providing a way to identify dysphagia, penetration, aspiration, and post-swallow residue [2,15]. However, the gold standard is not without limitations. VFSS procedures are considered costly due to the requirement of a radiology suite and multiple disciplinary staff. Accessibility is also a limiting factor in referring a patient for VFSS. Barriers may include acute or severe illness preventing travel to the suite, the remote location of the patient, or the absence of available instrumentation [16]. Another drawback of VFFS is the exposure to ionizing radiation for both patients and clinicians. The small amount of radiation exposure poses little risk for the average person. However, considering the co-morbidities that accompany a chronic illness and that dysphagia is a secondary condition, this population has an increased likelihood of medical procedures [4] and possible exposure.

Objective measures can quantify images from a VFSS and have removed many limitations of subjective interpretations through the quantification of the biomechanical events necessary for a functional swallow. These include but are not limited to the excursion and velocity of the hyoid bone and larynx, pharyngeal constriction, and timing/sequencing of movements [17,18,19]. Parameters such as hyoid bone and laryngeal excursion and velocity are often assessed and identified as critical components for functional swallowing [20]. Reduced elevation of the hyoid bone and larynx are common factors leading to decreased airway protection and consequently increased implications of aspiration [21,22]. However, these objective measurements are tedious and time-consuming, requiring imaging software and training. Due to these limitations, the clinical implementation of objective measures is low [23].

### 1.3. Alternative Approaches to Assessment

To overcome the limitations of VFSS, non-invasive means of hyoid excursion analysis have been developed by Donohue et al. (2021) using high-resolution cervical auscultation (HRCA) [24]. A similar study validated a mechano-acoustic device that can monitor respiratory activity and the occurrence of a swallow through movements and vibrations of the skin’s surface [25]. A small side experiment of the study employed an imaging technique, three-dimensional digital image correlation (3D-DIC), using two high-speed 2-megapixel cameras (at 500 fps). This side experiment produced skin displacements that distinguished signatures of respiratory and swallowing cycles and correlated with movements detected by the mechano-acoustic device. Displacement peaks were observed during swallows at the laryngeal prominence, which they expected was due to laryngeal excursion, yet this was not confirmed. The use of imaging methods to detect swallowing shows excellent potential for non-contact and remote assessment. However, this imaging experiment used only two subjects, and it was observed that the participants had a carbon spray over their necks. It is assumed that this is a drawback of the 3D-DIC method. Sakai et al. (2021) [26] produced a screening test for sarcopenic dysphagia with a static image of the anterior neck to characterize muscle wastage in the neck muscles, with the Features from Accelerated Segment Test (FAST) method. This study provides a non-invasive, non-contact method of dysphagia screening with the potential for remote assessment/screening for dysphagia. However, these methods are limited to the sarcopenic population.

Our group has previously employed image registration techniques to assess internal cardiac physiology [27]. A phase-based Savitzky–Golay gradient correlation (P-SG-GC) algorithm was able to precisely detect skin displacements produced by venous jugular pulse waveforms captured by a simple camera. When compared to existing algorithms, the P-SG-GC robustly performed better than existing methods on a variety of images and proved to be the most accurate, efficient, and robust algorithm for measuring in vivo skin deformations [28,29].

The current study applies image registration methods to the context of dysphagia screening to assess the feasibility of using similar methods for developing a dysphagia screening tool to detect at-risk patients. Skin displacements detected by a P-SG-GC algorithm and captured by iPhone cameras are predicted to be associated with hyolaryngeal excursions. The excursion of the hyoid bone and larynx, a mechanism of airway protection during swallowing, will theoretically cause displacements of the surrounding soft tissue and skin in these regions. The study examines the relationship between skin displacements, skin displacement velocity, hyolaryngeal excursion, and excursion velocity, and clinical measures of dysphagia severity in all patients referred for a VFSS at The Rose Centre, Christchurch, New Zealand. The clinical measures of penetration-aspiration and post-swallow residue can assess impaired swallowing safety and efficiency [30] and can be described by the Penetration-Aspiration Scale (PAS) [31], Residue Severity Ratings [32], and the Normalized Residue Ratio Scale [9].

## 2. Materials and Methods

### 2.1. Participants and Ethical Considerations

Thirty-one patients were referred to the Rose Centre for Stroke Recovery and Research for a videofluoroscopic swallowing assessment to evaluate pharyngeal swallowing between August 2021 and October 2022. All patients with symptoms of dysphagia were included if they had been referred for a VFSS at the Rose Centre. Patients were excluded if they had a history of any tracheal or laryngeal surgeries, pregnancy, barium allergies, or did not have sufficient ability to provide informed consent or maintain a seated position.

Twenty-three participants were included; eight were excluded due to neck surgeries (5), no capacity to provide informed consent (1), not providing consent for video recordings (1), and not being able to capture swallowing due to severe dysphagia (1). 92 recordings [23 patients × 4 swallows (2 × 3 mL and 2 × 20 mL)] were performed and due to data collection/recording errors, 79 sessions were analyzed for skin displacement/videofluoroscopy correlations and 61 for skin displacement/PAS & residue correlations. The patients included were of varying ages and etiologies, as displayed in Table 1. Age, gender, and diagnoses were collected from patients but were not considered in the study design or analyzed as this is a preliminary study. Informed consent was provided by all participants for study inclusion.

The study was conducted under the Declaration of Helsinki, approved by the University of Otago Health Human Ethics Committee on the 7 July 2021 (H21/094).

### 2.2. VFSS

The study was performed using a standard c-arm fluoroscopy unit (GE OEC Fluorostar 3, Wendelstein, Germany, 2014), with data recorded at 25 frames per second. The VFSS was performed by a speech and language therapist with significant clinical skill in dysphagia management who had undergone radiation training. Participants were seated as upright and still as possible. Patients received four separate barium contrast-thin liquids to swallow: two volumes of 3 mL and two of 20 mL. The 3 mL volumes were syringed directly into the patient’s mouth, either by the patient or a clinical assistant, depending on the patient’s ability. The 20 mL volumes were either provided in a single-use medicine cup or syringed directly into the mouth of the patient (not preferred by the patient). The patient was instructed to hold the liquid in the oral cavity until directed to swallow. The VFSS videos were recorded in the lateral plane, then stored for review. A 10-mm-wide ball bearing was taped to the participants’ chins to scale the recording for analysis. The videos obtained from the VFSS were used clinically and stored with the patient’s NHI number.

### 2.3. Video Recording

**Figure 1:**
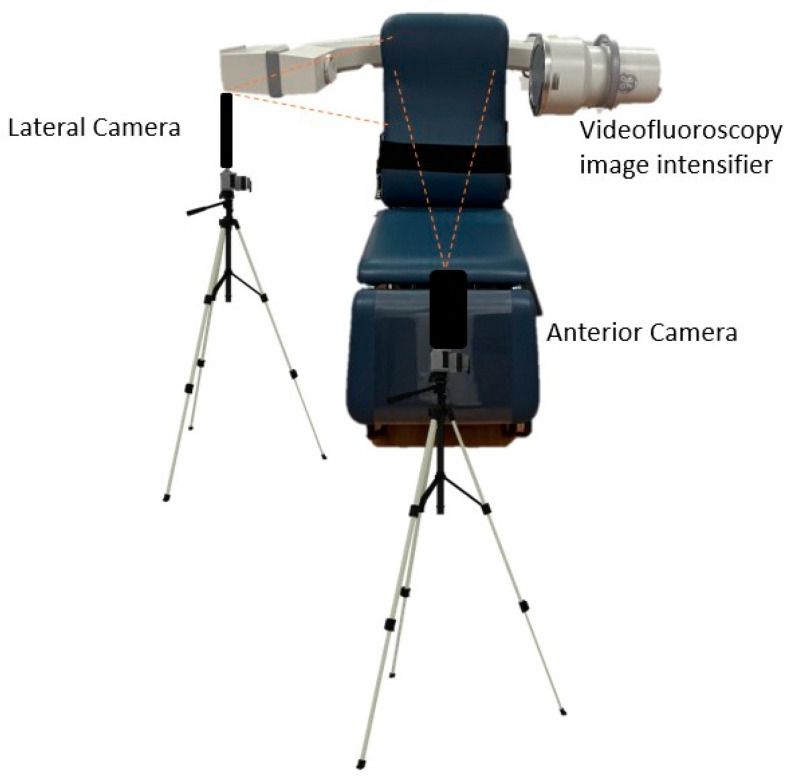
Placement of iPhones in comparison to the videofluoroscopy c-arm unit, where the patient would be seated in the chair. Please note that this is a digital model and is not to exact scale. Tripod image from https://lovepik.com/image-401271798/tripod.html (accessed on 5 May 2023).

Cameras and tripods were put into position once the patient was seated, and the fluoroscopy was positioned according to the patient’s height. Two iPhone X (Apple Cupertino USA) cameras were fixed on tripods approximately 0.5 m away from the patient. One iPhone camera was positioned to capture the lateral neck using the back-facing camera in the same plane as the VFSS image intensifier (Figure 1). This camera was recording in slow motion at 120 fps at 1080p. The second camera was positioned on a tripod to capture the anterior neck using the front-facing camera on the iPhone at 240 fps, 720 p. The cameras recorded simultaneously during the VFFS procedure.

The audio from the video recordings was used to synchronize with a distinct beep as the VFSS began recording. The audio waveforms of the video recording were processed using iMovie for accurate synchronization. The videos were converted to an .MOV file, and the front-facing video was also converted to 120 fps to be processed using the algorithm on MATLAB 2020.

### 2.4. Data Extraction

#### 2.4.1. Hyoid Bone Displacement

**Figure 2:**
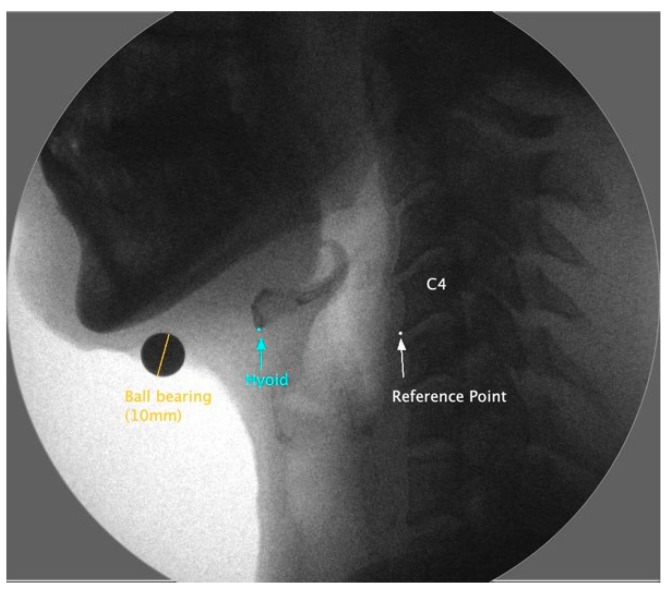
This is a lateral view of a videofluoroscopic frame of the resting hyoid bone (blue), showing the C4 frame reference point (white). The orange line illustrates the ball bearing length to which the measurements are scaled.

Frame-by-frame analysis determined the resting and maximum displacement positions of the hyoid bone. Still images were generated from the videos to capture these two positions for measurement. Using imaging software, ImageJ 1.53, the images were scaled using a ball bearing (10 mm) and analyzed to identify the coordinates of the most anterior and inferior point of C4 for a reference point and the most anterior and inferior corner of the hyoid bone, as seen in Figure 2.

The following equations were used to find anterior (horizontal) and superior (vertical) hyoid displacements using the coordinates [33]:

Horizontal displacement = (x2 − x1) − (C4x2 − C4x1)

Vertical displacement = (y2 − y1) − (C4y2 − C4y1)

where

x1 = resting horizontal coordinate of the hyoid bone;

x2 = displaced horizontal coordinate of hyoid bone;

y1 = resting vertical coordinate of hyoid bone;

y2 = displaced vertical coordinate of hyoid bone;

C4x1 and C4y1 = coordinates of vertebra C4 resting;

C4x2 and C4y2 = coordinates of C4 when hyoid is displaced.

The percentage of change in distance for both anterior and vertical distance was calculated using the formula:

% change = 100 × (hyoid displacement/hyoid resting)

#### 2.4.2. Hyoid Bone Displacement Velocity

Using the displacement distance percentage (%), the time taken to reach maximum displacement velocity (%/s) was calculated [34].

Velocity = distance change/time

#### 2.4.3. Laryngeal Excursion

**Figure 3:**
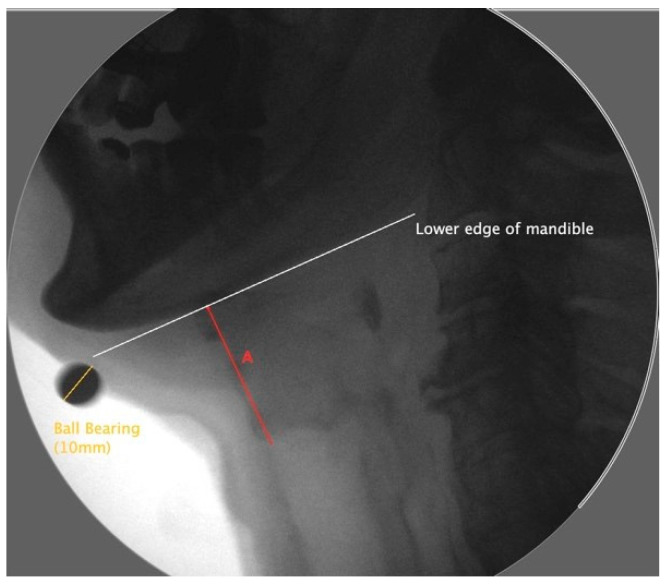
Lateral view of a videofluoroscopic frame displaying a maximally elevated larynx with a tangent line (white) to the mandible. Line A (red), perpendicular to the tangent line, is the measure between the larynx and mandible.

Frame-by-frame analysis was used to determine and produce still images of the laryngeal resting and maximum displacement positions. The images were analyzed and scaled using ImageJ. A line along the lowest edge of the mandible was used as a reference point. Measurements of the larynx were taken between the superior anterior corner of the air column and the reference line of the mandible. Laryngeal elevation was defined as the distance of the larynx and the mandible measured in the resting frame subtracted from the laryngeal mandible distance measured from the frame of maximal excursion, as shown in Figure 3 [34].

The percentage of change in distance was calculated using the formula [34]:

% change = 100 × (laryngeal displacement/laryngeal resting)

#### 2.4.4. Velocity of Laryngeal Elevation

The velocity of the maximum laryngeal elevation was calculated using the percentage of change in distance and time (%/s) [34].

Velocity = distance change/time

#### 2.4.5. Analysis of Aspiration

Aspiration was quantified using the PAS [31]. The 8-point scale gives points for the penetration depth of barium contrast with anatomical structures as landmarks.

#### 2.4.6. Residue Severity Ratings (RSV)

The videofluoroscopy recordings were analyzed frame-by-frame to determine when the hyoid had returned to the resting position after the first swallow. The Eisenhuber et al. (2002) [32] residue scale was used to categorize barium residue severity in the vallecula and pyriform sinuses. Categories were defined as none (0), mild (1), moderate (2), and severe (3), where mild was characterized as more than a thin coating, filling to a height of less than 25% of the structure, moderate filling between 25 and 50%, and severe residue was considered to fill over 50% of the structure.

#### 2.4.7. NRRS Residue Measures

**Figure 4:**
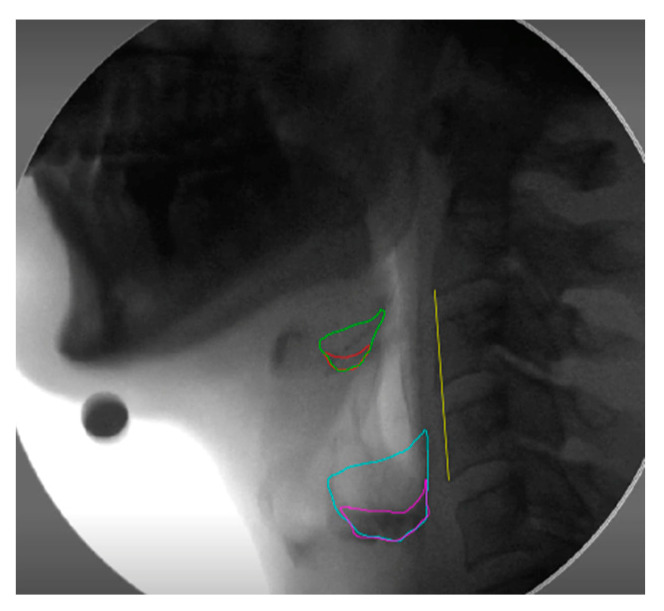
Lateral VFSS frame post-swallow. Outlined is the vallecular space (green), vallecular residue (red), pyriform sinus (blue), pyriform sinus residue (pink), and anatomical scalar C2-C4 (yellow).

The videofluoroscopy recordings were analyzed frame-by-frame to determine when the hyoid had returned to the resting position after the first swallow. A take on the normalized residue ratio scale (NRRS) [9] was incorporated in MATLAB to measure post-swallow residue in the vallecular space (v) and pyriform sinuses (p). The *imfreehand* and *imline* tools were used to outline the structural spaces, residue area, and C2-C4 scale length. The vallecular space was defined anteriorly by the perpendicular spine adjacent to the tip of the epiglottis and posteriorly by the epiglottis. The pyriform sinuses were defined using a line extending from the tip of the arytenoid shadow to the posterior pharyngeal wall, perpendicular to the vertebral axis. Each segmentation measured was overlaid on the final image (Figure 4), where the segmented areas and spinal distance were used to determine the NRRS values.

NRRSv
=
(A1/A1
+
A2)
×
[(A1/N2)
×
10]

where

A1 = Area of residue

A1 + A2 = Vallecular area

N2 = length of C2-C4

NRRSp =
(A1/A1
+
A2)
×
[(A1/N2)
×
10]

where

A1 = Area of residue

A1 + A2 = Pyriform sinus

N2 = length of C2-C4

### 2.5. Skin Displacements

**Figure 5:**
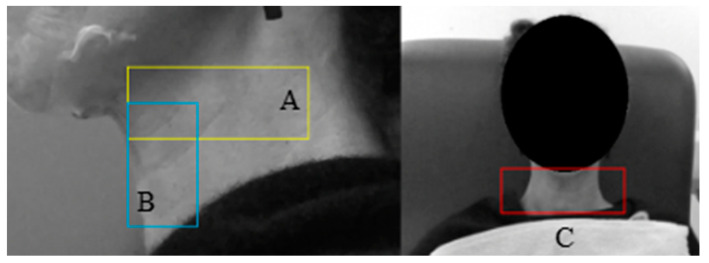
The external regions of analysis. Box A (yellow) represents the skin ROA for the hyoid bone; Box B (blue) represents the ROA for the larynx; and Box C (red) displays the anterior ROA.

The lateral and anterior videos were analyzed using the P-SG-GC algorithm [27] on MATLAB software to quantify the skin displacements during the swallow. Displacement vectors for different points in each video frame were extracted within the specified regions of interest (Figure 5) using the P-SG-GC image registration algorithm. The algorithm produces frames for the entire video. From those frames, 300 were used from a specified time point, aligning with the patient’s first swallow, confirmed by VFSS. From the specified 300 frames, skin displacement vectors were produced for a single region of analysis (ROA): A, B, or C (Figure 5).

**Figure 6:**
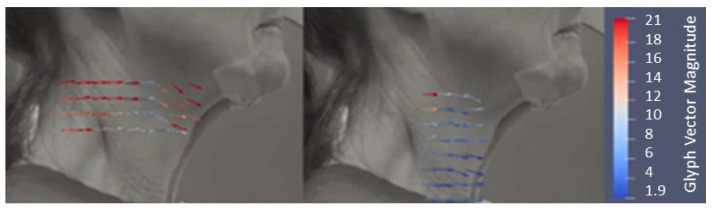
External visualization of skin displacement vectors (red and blue arrows). Left: skin displacements in the hyoid bone region of interest. Right: skin displacement in the laryngeal region of interest. Scale: a color scale representing the magnitude of skin displacement vectors.

The extracted vectors (Figure 6) were analyzed using ParaView and ImageJ to visualize and confirm the correct region of analysis with the visualization of the mean displacement vectors per frame.

The displacement vectors were exported into separate X and Y displacement points per frame. The vectors of each frame were then super-positioned to give the added displacement over time, so the displacement data is relative to the first frame. The vectors were then averaged and plotted on a scatter graph to visualize and select the minimum and maximum points of displacement. The scatter graph was used to calculate the overall displacement, time (converted from 120 fps to seconds), and velocity (%/s).

### 2.6. Inter-Rater Reliability

The primary researcher trained a second researcher in the VFSS measures of hyoid and laryngeal displacement, PAS scoring, the residual severity scale [32], and NRRS measures. Twenty percent of the data for each parameter was randomly selected for the inter-rater reliability measures, calculated using interclass correlation coefficients (ICCs).

### 2.7. Statistical Analysis

The study population consisted of dysphagic patients, so it was expected that the data would not be normally distributed. However, the Jarque–Bera normality test was used to assess the distribution. The variables were found to be both non-normally and normally distributed. The different variables were used to produce scatter plots and visualize outliers and the spread of the data. Due to the heterogeneous nature of swallows within individuals, non-independence was not accounted for by averaging swallows or other methods [35,36]. This is evidenced by Robbins et al. (1999) [37], who provide significant within-subject variation of PAS in patient populations. Spearman’s rank correlation coefficient was used due to the nature of the distribution of the data and the use of categorical measures (PAS and RSR). Correlation strengths were grouped as very weak (0.0–0.19), weak (0.2–0.39), moderate (0.40–0.59), strong (0.6–0.79), and very strong (0.8–1.0) [38]. Significance tests were performed on the Spearman rank correlation coefficient values using a two-tailed t-statistic test to produce a *p*-value. If the *p*-value was less than 0.05, it was concluded that there was sufficient evidence to accept the presence of a monotonic correlation between the variables analyzed. If the *p*-value exceeded 0.05, we concluded that there was insufficient evidence to indicate a correlation.

## 3. Results

### 3.1. Hyolaryngeal Excursion

Table 2 displays the Spearman’s rank correlation coefficients, a measure of the relationship between hyoid bone excursion in the anterior and superior directions, total laryngeal excursion, and skin displacements (captured by the lateral camera) in the horizontal (X) and vertical (Y) direction of the associated ROA. Please note that correlations between hyoid excursion and skin displacements were analyzed in the same plane e.g., horizontal skin displacement and anterior hyoid excursion are both in the X axis. Total laryngeal excursion was not further differentiated into anterior and superior components and was compared with both X and Y directional skin displacements. A strong correlation was observed between the anterior hyoid excursion and the horizontal (X) lateral skin displacement for a 20-mL bolus. The remaining correlation values of the hyolaryngeal excursion (anterior/superior) and the external displacements (vertical and horizontal) for both regions of analysis were statistically insignificant.

Skin displacement velocity, captured by the anterior camera, was moderately correlated with anterior hyoid excursion for a swallow of a 3 mL bolus, as displayed in Table 3. The remaining correlations between anteriorly captured skin displacement and hyolaryngeal excursion and velocity were insignificant.

### 3.2. Clinical Measures of Dysphagia Severity

#### 3.2.1. Penetration-Aspiration

Table 4 displays the Spearman’s rank correlation coefficients measuring the strength of the relationship between the hyoid, laryngeal and anterior ROAs (see Figure 5) and the PAS. The protocol specified that only participants who exhibited penetration/aspiration were included in further analysis to prevent skewed results. Four different groups were analyzed: all participants, those who exhibit some form of penetration with PAS scores of two and above, those who scored three and above, and those who penetrated only (no aspiration) with PAS scores of 2–5. Please note the small size of the group who scored three and above, hence why the scores were not further separated (e.g., 4+, 5+, 6+) as the numbers in the groups diminished. The table shows a very strong and significant correlation between vertical skin displacement velocity over the anterior ROA (anterior camera) and PAS scores of 3+. There were no significant correlations exhibited between skin displacement measures of the laryngeal region (lateral camera) and PAS scores. Moderate correlations were observed between the horizontal velocity of skin displacements of the hyoid ROA (lateral camera) and PAS scores of 2+ and 2–5. The *p*-value of these correlations was 0.053, slightly above the significance cut-off point for the study. However, these correlations should not be overlooked, as 0.3% is a small margin of error.

#### 3.2.2. Post-Swallow Residue

Table 5 displays the Spearman’s rank correlation coefficients measuring the strength of the relationship between the skin displacements over the hyoid, laryngeal, and anterior regions (See Figure 5) and residue measures using the RSR [32] and NRRS of the vallecula and pyriform sinus [9]. Those swallows with residue exhibited were analyzed separately from the group that included all swallows (both no residue and residue). NRRSp in those who had residue present was correlated with vertical skin displacements and vertical and horizontal skin displacement velocity over the hyoid ROA. There were strong and moderate correlations between NRRSp and laryngeal ROA X and Y directional skin displacements, respectively. The correlations between anterior ROA horizontal skin displacements and NRRSv in the groups that showed post-swallow residue were not statistically significant.

### 3.3. Inter-Rater Reliability

Table 6 displays the ICCs for the VFSS parameters, revealing excellent reliability for hyoid and laryngeal scoring, PAS, and residue ratings. The NRRS scores showed poor reliability.

## 4. Discussion

The study evaluated the feasibility of utilizing a smartphone camera to capture the relationship between skin displacements and biomechanical (hyoid and laryngeal excursion and velocity) and clinical measures (penetration-aspiration and post-swallow residue) associated with dysphagia. These evaluations are necessary for the development of a non-contact or remote dysphagia screening tool. A similar approach was employed by our group using the same base algorithm to successfully detect venous jugular pulse waveforms from skin displacements, assessing cardiac dysfunctions [27].

A strong correlation was observed between internal hyoid excursion and external skin displacements. This was specifically for a 20 mL bolus, anterior hyoid displacement, and horizontal skin displacement (hyoid ROA) captured by the lateral camera. Previous literature describes the larger and more consistent nature of anterior hyoid excursion in comparison to vertical excursion [39]. The consistent nature of the anterior displacement may have been a factor in detecting the significant correlation in the study’s smaller sample size. Another factor leading to this correlation may be that hyoid excursion is increased with increasing bolus size [40], so there are more likely increases in skin movement with a larger 20 mL bolus. The correlation seen between horizontal skin displacement velocity was captured by the anterior camera with a 3 mL bolus. It has been documented that with increased bolus sizes, hyoid excursion velocity increases [41,42]. For a larger velocity, there must be increased force due to increased muscle fiber activation. It is possible that with a smaller bolus, there were fewer muscle fiber activations and skin movements and, therefore, less noise.

The absence of other correlations between skin displacement and internal (hyoid and laryngeal) excursion could be due to the movement of multiple muscles altering the overall skin displacement vectors, disguising displacement related to hyolaryngeal movement. Multiple muscles in the neck are involved in coordinating a swallow or movement/stabilization [43]. The superficial musculoaponeurotic system (SMAS) is a network of collagen and elastin fibers and fat cells over the face and neck that embeds the muscles of the face and neck in the skin [44,45]. When the facial and neck muscles contract, the connected skin accompanies the muscle movement, producing a skin displacement, impacting indirect detection of hyolaryngeal displacement via the skin. As a result of the SMAS connection, the skin overlying the neck is more susceptible to movement and displacement with a swallow.

The suprahyoid and thyrohyoid muscles are responsible for hyolaryngeal elevation [46,47]. Firstly, these muscles have muscle fibers and bellies that are spread across the neck and have different attachment points over the mandible and cranial base [45]. When these muscles contract together for hyolaryngeal elevation, they contract in different directions, which may account for the observed noise. Secondly, the suprahyoid muscles are in the cervical fascia, connected to the SMAS. When these muscles contract, the connected skin accompanies the muscle movement, producing skin displacement.

Other muscle groups unrelated to hyolaryngeal excursion may also account for the observed results. There are many muscles in the neck, not involved in swallowing, that are required for head and neck stabilization and movement [48,49]. Many patients exhibited compensatory mechanisms required to swallow the presented bolus. These movements in the head and neck may have affected the muscles activated in the neck and altered the displacement points. The pharyngeal constrictor muscles (superior, medial, and inferior) are related to hyoid movement and play an essential role in swallowing [50]. Reduced pharyngeal constriction is associated with post-swallow residue [51,52], which may account for the correlations observed between skin displacements and measures of post-swallow residue.

Skin movements may reflect the culmination of the many coordinated events/movements measured in their entirety that make up the complex swallowing process. The results of the study may be interpreted purely as correlations between skin displacements and the overall effectiveness of the events occurring as assessed by clinical measures (PAS and post-swallow residue), as opposed to how the study was framed, assessing skin displacements of the hyoid region and laryngeal region. There is insufficient evidence to conclude that external measures of skin displacement are directly correlated with hyoid and laryngeal movement. However, the possibility of this relationship should not be ruled out. Due to clinical relevance, there were arguably more important correlations made between skin displacements and measures of impaired swallowing, including measures of aspiration and penetration as well as pharyngeal residual. These results indicate that the skin displacements could represent factors other than hyoid and laryngeal displacement related to impaired swallowing safety and efficiency.

Vertical skin displacement velocity, captured by the anterior camera, was very strongly correlated with PAS scores of three and above. The significance of this is that scores of 3+ are considered clinically pathological [53]. The algorithm detected skin displacements correlated with the risk of pathological penetration and aspiration scores. Importantly, this skin displacement was captured on the anterior-facing camera, which gives potential for future self-monitoring captured by the iPhone’s front-facing camera.

As opposed to the pathologically categorized (3+) scores, correlations were observed between horizontal skin displacement (hyoid region) and ranging scores of two and above on the PAS scale. The correlated scores included those exhibiting penetration and aspiration (2+) and penetrations only (2–5). This highlights that skin movement was correlated with swallowing events leading to material entering the larynx. The *p* values of the correlations for 2+ and 2–5 groups were 0.053, just above the stated significance cutoff point. The number of participants diminished when grouping PAS scores for analysis, which may have impacted significance scores. It is worth noting that patient safety was at the forefront of the study, so those at risk of severe aspiration were excluded from 20 mL swallows. This is likely to have influenced the incidence of aspiration, with only one patient exhibiting aspiration.

Penetration and aspiration have historically been associated with reduced hyoid displacement in dysphagic patients of various etiologies [54]. Those with reduced excursion are 3.7 times more likely to aspirate than those with normal excursion [20]. So, it is not unexpected that skin movement over the hyoid region is correlated with pathological PAS scores. Furthermore, the skin displacements correlated with PAS scores were in the horizontal direction. Anterior (horizontal) hyoid excursion has been indicated to be the only aspect of hyoid excursion predictive of penetration and aspiration risk [55] and significantly associated with the PAS [56]. Considering that the primary component between internal structural and skin displacement was anterior hyoid excursion and anterior skin displacement, this is a clinically significant finding. Inclusion of the penetration-only group is also relevant, as it has been documented that among those exhibiting deep laryngeal penetration, 85% go on to aspirate [57]. Additionally, laryngeal penetration has been associated with an increased incidence of aspiration pneumonia [58]. Therefore, the detection and monitoring of penetration, as shown to be possible in this study, is key to aiding in the prevention of aspiration and aspiration pneumonia.

Vertical skin displacement and velocity, as well as horizontal skin displacement velocity captured by the lateral camera, showed correlations with NRRSp. This coincides with prior studies indicating that reduced anterior hyoid movement is related to pyriform sinus residue in dysphagic patients [59,60]. Additionally, correlated with NRRSp was the horizontal skin displacements and velocity of the laryngeal region (lateral camera), with an additional relationship with pyriform sinus residue severity ratings. This is consistent with associations between reduced laryngeal excursion and impaired bolus clearance, resulting in post-swallow residue [60]. In contrast to the relationship between skin displacements and pyriform sinus residue, captured by the lateral camera, the anterior camera captured horizontal skin displacements that exhibited correlations with NRRSv, though not significant (*p* = 0.05). Post-swallow residue in either or both pharyngeal spaces is associated with an increased risk of penetration/aspiration. However, a study has found that with further analysis, only vallecular residue has been associated with decreased safety with the subsequent swallow [61]. Essentially, the presence of residue indicates reduced swallowing efficiency and an increased risk of penetration or aspiration [15,61].

Our results show there is potential for the algorithm to detect swallowing inefficiencies and increased risk using the skin displacements of both the hyoid and laryngeal regions. These skin displacements correlated with pharyngeal residue could be due to additional or other movements. For example, the pharyngeal constrictor muscles (superior, medial, and inferior) play an essential role in swallowing, with reduced constriction being associated with post-swallow residue (in both pharyngeal locations) and therefore an increased risk of penetration-aspiration [52]. Specifically, tongue-driving force is a factor associated with vallecular residue, whereas pharyngeal shortening is associated with pyriform sinus residue [62]. There are many factors that contribute to producing a safe and efficient swallow. For those with swallowing impairments, these factors may be predictors of penetration-aspiration or post-swallow residue. Further investigations are warranted to analyze the relationship between skin vectors and the long list of potential swallowing parameters associated with penetration-aspiration and pharyngeal residue [32,41,53,61,62,63,64,65,66,67,68,69].

The P-SG-GC algorithm detected skin displacement information indicative of potential swallowing dysfunction. Correlations with the PAS and the NRRS give insight into the most significant risk factors for dysphagia, and with use in the future, they may mark a patient with the need for further assessment and a more cautionary approach to eating and drinking. The skin displacements were obtained from videos captured by a mobile phone, demonstrating the capability of the accessible smartphone to detect the possible occurrence of penetration and post-swallow residue. There was little evidence to conclude that the skin displacement vectors produced were related specifically to hyoid or laryngeal movement. However, important correlations were produced between skin displacements and clinical measures of dysphagia severity assessed by the PAS and NRRS. This is a preliminary study that lays an excellent foundation for further investigations into other parameters related to swallowing safety and efficiency with a larger sample size. It would also be beneficial to investigate different etiologies, as this may have differing results due to different underlying physiologies contributing to swallowing impairments.

The study provides the potential for non-contact, non-invasive, and remote dysphagia screening and monitoring. This tool could benefit the dysphagic population by removing accessibility barriers such as limited mobility and cost. Because of the smartphone’s non-contact and widely available nature, there is also the potential to monitor the condition over time to assess the progression of the swallowing impairments, penetration, or residue. Patients may be evaluated in the comfort of their own environment or without disruptions. As the study was carried out using the camera on a mobile phone, there is the advantage of easy implementation as a mobile health (mHealth) application, a rapidly increasing field [70].

### Limitations

The primary purpose of this study was not to characterize swallowing, so patient etiologies and characteristics were not controlled for. Time constraints and restrictions on patients’ access to one center reduced the breadth of access to the population, limited the sample size, and may have increased selection bias, a factor to consider when interpreting results. A limitation of including a patient population was that swallowing strategies were performed by some to assist their swallowing, which may have led to discrepancies in skin displacement measures. Despite this, the inclusion of patients allowed for increased clinical application. The data collection coincided with standard clinical assessment, which means that internal validity may have been reduced, but the results are more generalizable and translatable to clinical practice. Camera positioning varied depending on patient height and the positioning of the VFSS machine. For future studies, it is recommended for the participants to hold the anterior facing camera in order to get a closer view of the skin. This is an initial feasibility study to investigate whether it is possible to detect skin displacements reflecting swallowing dysfunctions, so further validations and investigations are necessary. The current method of skin displacement analysis would not be directly used clinically due to the skills and steps required. Due to the potential shown in this study, there is further reason to continue the development of this screening tool. It is worth noting the diverse nature of the patient population and smaller sample size, which may impact the reproducibility of these results, yet we expect similar findings.

## 5. Conclusions

This study was the first in the development of a novel non-contact, non-invasive, and remote imaging method for evaluating oropharyngeal dysphagia using a smartphone. Skin displacements of the neck were correlated with swallowing dysfunctions of impaired safety and efficiency, as measured by PAS scores and the NRRS. Anterior hyoid excursion could also be correlated with skin displacements in the same plane with a 20-mL bolus. To our knowledge, this is the first study to capture indications of penetration and residue with a mobile phone using image registration methods to assess dysphagia.

Enhanced non-contact assessment and disease progression monitoring will reduce some of the many limitations associated with current methods, leading to early and accurate treatment. Ultimately, enhanced assessment methods would contribute to decreasing dysphagic patients’ risk for potentially life-threatening co-morbidities, anxieties, and strain on the health care system.

Future studies should focus on including other swallowing parameters indicative of dysphagia with the incorporation of surface electromyography (sEMG) to provide additional insight into muscle activations that may also be associated with skin displacements.

## Figures and Tables

**Table 1:** Patient characteristics.

Age	20–80 years
Sex	11 Male, 12 Female
Diagnosis	Huntington gene positive (2); inclusion body myositis (1); esophageal motility disorder (1); diffuse idiopathic skeletal hyperostosis (1); stroke (3); listeria rhombencephalitis (1); muscular dystrophy (1); traumatic brain injury (2); head and neck cancer (2); cerebral palsy (1); Parkinson’s disease (1); multiple sclerosis (1); rigid spine (1); diagnosis unknown (5).

**Table 2:** Spearman’s rank correlations (*p* value) of lateral camera-captured skin displacements of the hyoid and laryngeal ROAs vs. hyoid and laryngeal excursion and excursion velocity.

	Hyoid		Larynx	
Skin Displacements of Corresponding ROA	3 mL	20 mL	3 mL	20 mL
X displacement (%)	−0.25 (0.11)	0.67 *** (0.0001)	0.08 (0.6)	0.17 (0.42)
Y displacement (%)	0.07 (0.66)	0.04 (0.81)	0.17 (0.29)	0.34 (0.09)
Xdisplacement velocity (%/s)	−0.21 (0.20)	0.14 (0.48)	0.29 (0.07)	−0.34 (0.09)
Y displacement velocity (%/s)	0.22 (0.16)	−0.30 (0.12)	0.04 (0.8)	−0.11 (0.72)

Note: Significant correlations with *p* < 0.05 are displayed as *** = strong (0.6–0.79).

**Table 3:** Spearman’s rank correlations (*p* values) of hyolaryngeal excursion and excursion velocity and skin displacements and displacement velocity, captured by the anterior camera.

	Hyoid		Larynx	
Skin Displacements of Corresponding ROA	3 mL	20 mL	3 mL	20 mL
X displacement (%)	−0.06 (0.77)	−0.05 (0.85)	0.01 (0.92)	−0.002 (0.99)
Y displacement (%)	−0.15 (0.35)	0.02 (0.9)	0.27 (0.09)	−0.18 (0.42)
X displacement velocity (%/s)	−0.44 ** (0.04)	−0.16 (0.61)	0.023 (0.90)	−0.37 (0.11)
Y displacement velocity (%/s)	−0.15 (0.35)	0.02 (0.90)	0.23 (0.18)	−0.05 (0.81)

Note: Significant correlations with *p* < 0.05 are displayed as ** = moderate (0.40–0.59).

**Table 4:** Spearman’s rank correlations (*p values*) of skin displacements over the hyoid, laryngeal, and anterior ROAs and PAS scores.

	Skin Displacements	PAS (all n = 61)	PAS (scores of 2+) (n = 26)	PAS (3+) (n = 11)	PAS (2–5, Penetration only = 25)
	X displacement (%)	0.16 (0.23)	0.35 (0.09)	0.33 (0.34)	0.35 (0.09)
Hyoid ROA	Y displacement (%)	−0.06 (0.65)	0.02 (0.92)	0.31 (0.35)	0.02 (0.9)
	X velocity (%/s)	0.13 (0.31)	0.40 (0.053)	0.58 (0.07)	0.41 (0.053)
	Y velocity (%/s)	−0.07 (0.56)	0.06 (0.76)	0.48 (0.15)	0.06 (0.76)
Larynx ROA	X displacement (%)	0.02 (0.88)	0.53 (0.09)	0.77 (0.22)	0.53 (0.09)
	Y displacement (%)	−0.09 (0.54)	0.39 (0.23)	0.97 (0.78)	0.39 (0.23)
	X velocity (%/s)	−0.09 (0.55)	0.30 (0.37)	0.77 (0.22)	0.3 (0.37)
	Y velocity (%/s)	−0.09 (0.71)	−0.08 (0.80)	−0.44 (0.55)	−0.08 (0.80)
Anterior ROA	X displacement (%)	0.100 (0.47)	0.18 (0.48)	0.55 (0.12)	0.18 (0.48)
	Y displacement (%)	0.07 (0.62)	0.15 (0.56)	−0.56 (0.14)	0.15 (0.56)
	X velocity (%/s)	0.12 (0.39)	0.14 (0.56)	0.42 (0.29)	0.14 (0.56)
	Y velocity (%/s)	0.09 (0.51)	0.16 (0.51)	−0.80 *** (0.01)	0.17 (0.51)

Note: Significant correlations with *p* < 0.05 are displayed as *** = very strong (0.8–1.0). PAS = Penetration-Aspiration Scale.

**Table 5:** Spearman’s rank correlation (*p* value) of skin displacements over the hyoid, laryngeal and anterior ROAs vs. post-swallow residual measures (NRRS and RSR).

	Skin Displacements	RSRv (n = 61)	RSRp (n = 61)	NRRS v (n = 61)	NRRS p (n = 61)	RSRv (Exhibit Residue n = 37)	RSRp (Exhibit Residue n = 37)	NRRS v (Exhibit Residue n = 37)	NRRS p (Exhibit Residue n = 37)
Hyoid ROA	X displacement (%)	−0.22 (0.08)	−0.10 (0.43)	−0.08 (0.52)	−0.06 (0.65)	−0.20 (0.26)	−0.08 (0.57)	−0.34 (0.22)	−0.05 (0.78)
	Y displacement (%)	0.01 (0.91)	0.03 (0.83)	0.06 (0.64)	0.01 (0.93)	−0.01 (0.53)	0.07 (0.66)	−0.21 (0.41)	0.41 ** (0.047)
	X velocity (%/s)	−0.16 (0.20)	−0.08 (0.53)	−0.15 (0.23)	−0.11 (0.4)	−0.11 (0.40)	0.01 (0.53)	−0.27 (0.34)	0.47 ** (0.010)
	Y velocity (%/s)	0.05 (0.70)	0.06 (0.62)	0.06 (0.64)	0.03 (0.47)	−0.12 (0.80)	0.15 (0.31)	−0.13 (0.59)	0.57 ** (0.001)
Larynx ROA	X displacement (%)	0.04 (0.77)	0.04 (0.79)	−0.23 (0.13)	0.004 (0.77)	−0.04 (0.83)	−0.33 * (0.04)	−0.33 (0.28)	0.62 *** (0.03)
	Y displacement (%)	−0.22 (0.12)	−0.13 (0.38)	−0.15 (0.29)	−0.17 (0.22)	−0.11 (0.55)	−0.32 (0.05)	−0.32 (0.29)	−0.48 ** (0.03)
	X velocity (%/s)	0.15 (0.31)	0.07 (0.65)	−0.16 (0.30)	0.07 (0.66)	0.04 (0.85)	−0.23 (0.18)	−0.10 (0.74)	−0.05 (0.83)
	Y velocity (%/s)	−0.11 (0.47)	−0.02 (0.87)	0.0009 (0.99)	0.01 (0.9)	0.13 (0.50)	−0.23 (0.16)	−0.32 (0.31)	−0.17 (0.2)
Anterior ROA	X displacement (%)	−0.16 (0.23)	−0.08 (0.58)	0.11 (0.49)	0.07 (0.64)	−0.32 (0.07)	−0.01 (0.55)	−0.45 (0.050)	0.37 (0.16)
	Y displacement (%)	−0.0782 (0.57)	0.123 (0.36)	0.03 (0.82)	0.04 (0.77)	0.05 (0.74)	0.08 (0.59)	−0.20 (0.53)	−0.08 (0.74)
	X velocity (%/s)	−0.13 (0.33)	0.03 (0.82)	0.10 (0.54)	0.05 (0.73)	−0.28 (0.12)	0.009 (0.95)	−0.36 (0.16)	0.46 (0.07)
	Y velocity (%/s)	−0.008 (0.95)	0.17 (0.22)	0.07 (0.65)	0.04 (0.79)	0.12 (0.50)	0.057 (0.72)	−0.26 (0.43)	−0.17 (0.52)

Note: Significant correlations with *p* < 0.05 are displayed as * = weak (0.2–0.39), ** = moderate (0.40–0.59), *** = strong (0.6–0.79). RSRv—residue severity ratings of the vallecula; RSRp—residue severity ratings of the piriform sinus; NRRSv—normalized residue ratio of the vallecula; NRRSp—normalized residue ratio of the piriform sinus.

**Table 6:** Inter-rater reliability calculated as an interclass correlation coefficient (ICC).

Parameters	ICC Value	Interpretation
Internal Hyoid displacement	0.95	Excellent reliability
Internal Laryngeal displacement	0.97	Excellent reliability
PAS rating	0.99	Excellent reliability
Residue Severity ratings	0.97	Excellent reliability
NRRSv	0.34	Poor reliability
NRRSp	0.23	Poor reliability

## Data Availability

The data availability is limited (based on the ethical approval conditions).

## References

[1] Carucci L.R., Turner M.A. (2015). Dysphagia revisited: Common and unusual causes. RadioGraphics.

[2] Cohen D.L., Roffe C., Beavan J., Blackett B., Fairfield C.A., Hamdy S., Bath P.M. (2016). Post-stroke dysphagia: A review and design considerations for future trials. Int. J. Stroke.

[3] Medin J., Larson J., von Arbin M., Wredling R., Tham K. (2010). Elderly persons’ experience and management of eating situations 6 months after stroke. Disabil. Rehabil..

[4] Altman K.W., Yu G.P., Schaefer S.D. (2010). Consequence of dysphagia in the hospitalized patient: Impact on prognosis and hospital resources. Arch. Otolaryngol. Head Neck Surg..

[5] Patel D.A., Krishnaswami S., Steger E., Conover E., Vaezi M.F., Ciucci M.R., Francis D.O. (2017). Economic and survival burden of dysphagia among inpatients in the United States. Dis. Esophagus.

[6] Nguyen N.P., Frank C., Moltz C.C., Vos P., Smith H.J., Karlsson U., Sallah S. (2005). Impact of dysphagia on quality of life after treatment of head-and-neck cancer. Int. J. Radiat. Oncol. Biol. Phys..

[7] Wilson R.D. (2012). Mortality and cost of pneumonia after stroke for different risk groups. J. Stroke Cerebrovasc. Dis..

[8] Feng M.C., Lin Y.C., Chang Y.H., Chen C.H., Chiang H.C., Huang L.C., Hung C.H. (2019). The mortality and the risk of aspiration pneumonia related with dysphagia in Stroke Patients. J. Stroke Cerebrovasc. Dis..

[9] Pearson W.G., Molfenter S.M., Smith Z.M., Steele C.M. (2013). Image-based measurement of post-swallow residue: The normalized residue ratio scale. Dysphagia.

[10] Carbo A., Brown M., Nakrour N. (2021). Fluoroscopic Swallowing Examination: Radiologic Findings and Analysis of Their Causes and Pathophysiologic Mechanisms. RadioGraphics.

[11] Lind C.D. (2003). Dysphagia: Evaluation and treatment. Gastroenterol. Clin. N. Am..

[12] Deborah J.C., Ramsey M.R.C.P., David G., Smithard M.D., Lalit K. (2003). Early Assessments of Dysphagia and Aspiration Risk in Acute Stroke Patients. Stroke.

[13] Guan X.-L., Wang H., Huang H.-S., Meng L. (2015). Prevalence of dysphagia in multiple sclerosis: A systematic review and meta-analysis. Neurol. Sci..

[14] Wilson R.D., Howe E.C. (2012). A cost-effectiveness analysis of screening methods for dysphagia after stroke. PMR.

[15] Yoon J.A., Kim S.H., Jang M.H., Kim S.D., Shin Y.B. (2019). Correlations between aspiration and pharyngeal residue scale scores for fibreoptic endoscopic evaluation and videofluoroscopy. Yonsei Med. J..

[16] Rumbach A., Coombes C., Doeltgen S. (2017). A survey of australian dysphagia practice patterns. Dysphagia.

[17] Kendall K., McKenzie S., Leonard R. (2000). Timing of Events in Normal Swallowing: A Videofluoroscopic Study. Dysphagia.

[18] Choi K.H., Ryu J.S., Kim M.Y. (2011). Kinematic Analysis of Dysphagia: Significant Parameters of Aspiration Related to Bolus Viscosity. Dysphagia.

[19] Leonard R., Kendall K., McKenzie S. (2000). Structural Displacements in Normal Swallowing: A Videofluoroscopic Study. Dysphagia.

[20] Perlman A.L., Booth B., Grayhack J. (1994). Videofluoroscopic predictors of aspiration in patients with oropharyngeal dysphagia. Dysphagia.

[21] Cichero J., Murdoch B. (2006). Applied anatomy and physiology of the normal swallow. Dysphagia: Foundation, Theory and Practice.

[22] Hsiao M.Y., Chang Y.C., Chen W.S., Chang H.-Y., Wang T.-G. (2012). Application of ultrasonography in assessing oropharyngeal dysphagia in stroke patients. Ultrasound Med. Biol..

[23] Nordin N.A., Miles A., Allen J. (2017). Measuring competency development in objective evaluation of videofluoroscopic swallowing studies. Dysphagia.

[24] Donohue C., Khalifa Y., Perera S., Sejdić E., Coyle J.L. (2021). A Preliminary Investigation of Whether HRCA Signals Can Differentiate Between Swallows from Healthy People and Swallows from People with Neurodegenerative Diseases. Dysphagia.

[25] Kang Y.J., Arafa H.M., Yoo J.Y. (2022). Soft skin-interfaced mechano-acoustic sensors for real-time monitoring and patient feedback on respiratory and swallowing biomechanics. NPJ Digit. Med..

[26] Sakai K., Gilmour S., Hoshino E., Nakayama E., Momosaki R., Sakata N., Yoneoka D. (2021). A machine learning-based screening test for sarcopenic dysphagia using image recognition. Nutrients.

[27] Lam Po Tang E.J., HajiRassouliha A., Nash M.P., Nielsen P.M.F., Taberner A.J., Cakmak Y.O. (2018). Non-contact quantification of jugular venous pulse waveforms from skin displacements. Sci. Rep..

[28] HajiRassouliha A., Taberner A.J., Nash M.P., Nielsen P.M.F., Wittek A., Joldes G., Nielsen P., Doyle B., Miller K. (2017). Subpixel measurement of living skin deformation using intrinsic features. Computational Biomechanics for Medicine.

[29] HajiRassouliha A., Taberner A.J., Nash M.P., Nielsen P.M.F. (2018). Subpixel phase-based image registration using Savitzky–Golay differentiators in gradient-correlation. Comput. Vis. Image Underst..

[30] Molfenter S.M., Brates D., Herzberg E., Noorani M., Lazarus C. (2018). The swallowing profile of healthy aging adults: Comparing noninvasive swallow tests to videofluoroscopic measures of safety and efficiency. J. Speech Lang. Hear. Res..

[31] Rosenbek J.C., Robbins J.A., Roecker E.B., Coyle J.L., Wood J.L. (1996). A penetration-aspiration scale. Dysphagia.

[32] Eisenhuber E., Schima W., Schober E., Pokieser P., Stadler A., Scharitzer M., Oschatz E. (2002). Videofluoroscopic assessment of patients with dysphagia. Am. J. Roentgenol..

[33] Kim Y., McCullough G.H. (2010). Maximal hyoid excursion in poststroke patients. Dysphagia.

[34] Zhang J., Zhou Y., Wei N., Yang B., Wang A., Zhou H., Groher M. (2016). Laryngeal elevation velocity and aspiration in acute ischemic stroke patients. PLoS ONE.

[35] Cullins M.J., Gill J.P., McManus J.M., Lu H., Shaw K.M., Chiel H.J. (2015). Sensory feedback reduces individuality by increasing variability within Subjects. Curr. Biol..

[36] Söder N., Miller N. (2002). Using ultrasound to investigate intrapersonal variability in durational aspects of tongue movement during swallowing. Dysphagia.

[37] Robbins J., Coyle J., Rosenbek J., Roecker E., Wood J. (1999). Differentiation of normal and abnormal airway protection during swallowing using the penetration–aspiration scale. Dysphagia.

[38] Wechsler S., Campbell M.J.T., Swinscow D.V. (1996). Statistics at Square One.

[39] Ishida R., Palmer J.B., Hiiemae K.M. (2002). Hyoid motion during swallowing: Factors affecting forward and upward displacement. Dysphagia.

[40] Molfenter S.M., Steele C.M. (2011). Physiological variability in the deglutition literature: Hyoid and laryngeal kinematics. Dysphagia.

[41] Steele C.M., Cichero J.A.Y. (2014). Physiological factors related to aspiration risk: A systematic review. Dysphagia.

[42] Ueda N., Nohara K., Kotani Y., Tanaka N., Okuno K., Sakai T. (2013). Effects of the bolus volume on hyoid movements in normal individuals. J. Oral. Rehabil..

[43] Ertekin C., Aydogdu I. (2003). Neurophysiology of swallowing. Clin. Neurophysiol..

[44] Warshafsky D., Goldenberg D., Kanekar S.G. (2012). Imaging anatomy of deep neck spaces. Otolaryngol. Clin. N. Am..

[45] Mitz V., Peyronie M. (1976). The superficial musculo-aponeurotic system (SMAS) in the parotid and cheek area. Plast. Reconst. Surg..

[46] Humbert I.A., Poletto C.J., Saxon K.G., Kearney P.R., Crujido L., Wright-Harp W., Ludlow C.L. (2006). The effect of surface electrical stimulation on hyolaryngeal movement in normal individuals at rest and during swallowing. J. Appl. Physiol..

[47] Pearson W.G., Langmore S.E., Zumwalt A.C. (2011). Evaluating the structural properties of suprahyoid muscles and their potential for moving the hyoid. Dysphagia.

[48] Jung B., Bhutta S. (2022). Anatomy, Head and Neck, Neck Movements. StatPearls.

[49] Kohan E.J., Wirth G.A. (2014). Anatomy of the neck. Clin. Plast. Surg..

[50] Malone J.C., Arya N.R. (2022). Anatomy, Head and Neck, Swallowing. StatPearls.

[51] Leonard R. (2019). Predicting aspiration risk in patients with dysphagia: Evidence from fluoroscopy. Laryngoscope Investig. Otolaryngol..

[52] Stokely S.L., Peladeau-Pigeon M., Leigh C., Molfenter S.M., Steele C.M. (2015). The relationship between pharyngeal constriction and post-swallow residue. Dysphagia.

[53] Li B., Zhang T., Sun X., Xu J., Jiang G. (2010). Quantitative videofluoroscopic analysis of penetration-aspiration in post-stroke patients. Neurol. India.

[54] Perlman A.L., VanDaele D.J., Otterbacher M.S. (1995). Quantitative assessment of Hyoid bone displacement from video images during swallowing. J. Speech Lang. Hear. Res..

[55] Zhang Z., Perera S., Donohue C., Kurosu A., Mahoney A.S., Coyle J.L., Sejdić E. (2020). The prediction of risk of penetration–aspiration via hyoid bone displacement features. Dysphagia.

[56] Zhang Z., Kurosu A., Coyle J.L., Perera S., Sejdić E. (2021). A generalized equation approach for hyoid bone displacement and penetration–aspiration scale analysis. SN Appl. Sci..

[57] Friedman B., Frazier J.B. (2000). Deep laryngeal penetration as a predictor of aspiration. Dysphagia.

[58] Gurberg J., Birnbaum R., Daniel S.J. (2015). Laryngeal penetration on videofluoroscopic swallowing study is associated with increased pneumonia in children. Int. J. Pediatr. Otorhinolaryngol..

[59] Nakamura T., Kita Y., Fujimoto J., Ayuzawa K., Ozawa H. (2021). Hyoid bone movement during swallowing and mechanism of pharyngeal residue in patients with profound intellectual and multiple disabilities. Int. J. Pediatr. Otorhinolaryngol..

[60] Steele C.M., Bailey G.L., Chau T., Molfenter S.M., Oshalla M., Waito A.A., Zoratto D.C. (2011). The relationship between hyoid and laryngeal displacement and swallowing impairment. Clin. Otolaryngol..

[61] Molfenter S.M., Steele C.M. (2013). The relationship between residue and aspiration on the subsequent swallow: An application of the normalized residue ratio scale. Dysphagia.

[62] Dejaeger E., Pelemans W., Ponette E., Joosten E. (1997). Mechanisms involved in postdeglutition retention in the elderly. Dysphagia.

[63] Troche M.S., Huebner I., Rosenbek J.C., Okun M.S., Sapienza C.M. (2011). Respiratory-swallowing coordination and swallowing safety in patients with parkinson’s disease. Dysphagia.

[64] Ellerston J.K., Heller A.C., Houtz D.R., Kendall K.A. (2016). Quantitative measures of swallowing deficits in patients with parkinson’s disease. Ann. Otol. Rhinol..

[65] Han T.R., Paik N.-J., Park J.W. (2001). Quantifying swallowing function after stroke: A functional dysphagia scale based on videofluoroscopic studies. Arch. Phys. Med. Rehabil..

[66] Gaeckle M., Domahs F., Kartmann A., Tomandl B., Frank U. (2019). Predictors of penetration-aspiration in parkinson’s disease patients with dysphagia: A retrospective analysis. Ann. Otol. Rhinol. Laryngol..

[67] Han H., Shin G., Jun A., Park T., Ko D., Choi E., Kim Y. (2016). The relation between the presence of aspiration or penetration and the clinical indicators of dysphagia in poststroke survivors. Ann. Rehabil. Med..

[68] Curtis J.A., Molfenter S., Troche M.S. (2020). Predictors of residue and airway invasion in parkinson’s disease. Dysphagia.

[69] Rofes L., Arreola V., Romea M., Palomera E., Almirall J., Cabré M., Serra-Prat M., Clavé P. (2010). Pathophysiology of oropharyngeal dysphagia in the frail. J. Neurogastroenterol. Motil..

[70] Tarricone R., Petracca F., Ciani O., Cucciniello M. (2021). Distinguishing features in the assessment of mHealth apps. Expert. Rev. Pharmacoecon. Outcomes Res..

